# Preconditioning of umbilical cord‐derived mesenchymal stem cells by rapamycin increases cell migration and ameliorates liver ischaemia/reperfusion injury in mice via the CXCR4/CXCL12 axis

**DOI:** 10.1111/cpr.12546

**Published:** 2018-12-10

**Authors:** Jun Zheng, Hui Li, Liying He, Yiming Huang, Jianye Cai, Liang Chen, Chaorong Zhou, Hongyuan Fu, Tongyu Lu, Yingcai Zhang, Jia Yao, Yang Yang

**Affiliations:** ^1^ Department of Hepatic Surgery and Liver Transplantation Center the Third Affiliated Hospital of Sun Yat‐sen University Guangzhou China; ^2^ Organ Transplantation Research Center of Guangdong Province Guangzhou China; ^3^ Guangdong Key Laboratory of Liver Disease Research, Key Laboratory of Liver Disease Biotherapy and Translational Medicine of Guangdong Higher Education Institutes the Third Affiliated Hospital of Sun Yat‐sen University Guangzhou China; ^4^ Department of Gastroenterology Guangzhou Women and Children's Medical Center Guangzhou China

**Keywords:** autophagy, CXCR4, liver ischaemia/reperfusion injury, migration, preconditioning, umbilical cord‐derived mesenchymal stem cells

## Abstract

**Objectives:**

Transfusion of umbilical cord‐derived mesenchymal stem cells (UC‐MSCs) is a novel strategy for treatment of various liver diseases. However, the therapeutic effect of UC‐MSCs is limited because only a few UC‐MSCs migrate towards the damaged regions. In this study, we observed the effects of autophagy on the migration of UC‐MSCs in vitro and in a model of liver ischaemia/reperfusion (I/R) injury.

**Materials and Methods:**

We investigated the effects of autophagy on the status of the cell, release of anti‐inflammatory factors and migration of UC‐MSCs in vitro. The therapeutic effects and in vivo migration of rapamycin‐preconditioned UC‐MSCs were observed in a C57/B6 mouse model of liver I/R injury.

**Results:**

Induction of autophagy by rapamycin enhanced the ability of UC‐MSCs to migrate and release anti‐inflammatory cytokines as well as increased expression of CXCR4 without affecting cell viability. Inhibition of CXCR4 activation markedly decreased migration of these cells. In a mouse model of liver I/R injury, we found significantly upregulated expression of CXCR12 in the damaged liver. More rapamycin‐preconditioned UC‐MSCs migrated towards the ischaemic regions than 3‐methyladenine‐preconditioned or non‐preconditioned UC‐MSCs, leading to improvement in hepatic performance, pathological changes and levels of inflammatory cytokines. These effects were abolished by AMD3100.

**Conclusions:**

Preconditioning of UC‐MSCs by rapamycin afforded increased protection against liver I/R injury by enhancing immunosuppression and strengthening the homing and migratory capacity of these cells via the CXCR4/CXCL12 axis.

## 1 INTRODUCTION

Liver ischaemia/reperfusion (I/R) injury is a complicated pathophysiological process that can lead to initial poor function or primary non‐function of the liver as well as increased morbidity and mortality after hepatectomy and liver transplantation.^1^ The pathological characteristics in the ischaemic period are depletion of ATP and metabolic disturbance, whereas accumulation of reactive oxygen species (ROS), upregulation of cytokines and inflammatory cell infiltration occur after reperfusion; these changes induce further necrosis and apoptosis of hepatocytes.^1^, ^2^ Therefore, potential therapeutic strategies are urgently needed to reduce hepatic I/R injury and promote regeneration of hepatocytes.

Mesenchymal stem cells (MSCs) have a recognized use in cell‐based therapy given their ability to modulate inflammation and improve tissue regeneration and their low immunogenicity.^3^ The potential applications of MSCs have been explored in a number of diseases associated with organ dysfunction and disorders of the immune system, such as acute liver failure,^4^ Crohn's disease^5^ and graft‐versus‐host disease.^6^ Previous studies by our group and by other researchers have demonstrated that MSCs are ideal candidates for treating liver I/R injury.^7^, ^8^ However, the beneficial role of MSCs is limited because most exogenous MSCs are sequestered in the lungs and few are permitted to migrate and engraft the damaged tissues.^9^, ^10^ Therefore, efficient homing of MSCs towards tissue‐specific conditions is one of the most important factors in successful clinical therapy.

A mechanism that might strengthen the migratory capacity of exogenously transplanted UC‐MSCs involves targeting of the chemokine (C‐X‐C motif) ligand 12 (CXCL12)/chemokine (C‐X‐C motif) receptor 4 (CXCR4) axis.^11^, ^12^ It is well known that expression of CXCL12 is markedly increased in injured tissues, including the liver, brain and kidney^13^, ^14^, ^15^ and contributes to recruitment of CXCR4‐positive cells. However, only a small proportion of MSCs express CXCR4 during in vitro expansion, so their capacity to respond to homing signals in damaged regions may be limited.^12^ Therefore, targeting CXCR4 may improve the migratory and therapeutic efficiency of MSCs. Various strategies have been proposed to increase the expression of CXCR4 in MSCs. Although genetic and enzymatic modifications are available for manipulation of MSCs, there are still several safety concerns regarding their possible effects on cellular function and viability.^16^ Many recent studies have focused on the development of novel strategies for preconditioning of MSCs using hypoxic conditions, chemical compounds and cytokines during ex vivo expansion.^17^ And several previous studies have reported hypoxia‐preconditioned MSCs not only played a beneficial effect on attenuating acute kidney injury via enhancing the ability of angiogenesis and anti‐oxidation, but also strengthened the therapeutic effects for renal I/R injury through upregulated SDF‐1‐CXCR4/CXCR7 axis and chemotaxis.^18^, ^19^ On the other side, Witte showed that pre‐treatment with various cytokines was benefit for UC‐MSCs to treat inflammatory liver disease by promoting their immunomodulatory capacity,^20^ and Dang et al demonstrated that treated with cytokines before application could enhance the roles of MSCs in improving experimental autoimmune encephalomyelitis via upregulating their immunoregulatory function.^21^

Rapamycin is produced by *Streptomyces hygroscopicus* and is used clinically as an antifungal treatment and for immunoregulation.^22^, ^23^ However, rapamycin has also been reported to activate autophagy via inhibition of the mammalian target of rapamycin complex.^24^ Autophagy is a crucial and conserved biological degradation process that continuously degrades dysfunctional organelles and abnormal proteins to maintain cellular stability and activity under different pathological and physiological conditions.^25^ Accumulating evidence suggests that different cell milieus, including hypoxia, starvation, inflammation and hyperpyrexia, can alter autophagy.^25^, ^26^ Furthermore, some studies have shown a correlation between autophagy in MSCs and immunosuppression.^21^, ^27^ However, it is unclear whether induction of autophagy would strengthen homing of MSCs to damaged liver tissue or have a therapeutic effect in liver I/R injury.

Therefore, the aim of the present study was to investigate whether induction of autophagy by rapamycin would influence survival of umbilical cord‐derived (UC)‐MSCs in terms of their immunoregulatory and migratory ability. We also evaluated the efficiency of rapamycin‐treated UC‐MSCs in an in vivo liver I/R injury model.

## 2 MATERIALS AND METHODS

### 2.1 Isolation, culture and characterization of UC‐MSCs

The human fresh UCs from which MSCs were isolated were collected from 10 healthy donors after they had provided written informed consent. All procedures were approved by the Research Ethics Committee of the Third Affiliated Hospital of Sun Yat‐sen University and performed under aseptic and standardized conditions. UC‐MSCs were isolated and cultured as described previously.^28^ In brief, the fresh UCs were collected after birth and submerged in phosphate‐buffered saline (PBS). After being washed carefully with PBS to remove the residual blood, the UCs were cut into 10‐mm^3^ pieces and placed in a solution containing collagenase type 1 (Gibco, Gaithersburg, MD) with 0.1% hyaluronidase (Sigma‐Aldrich, St Louis, MO) and 3 mmol/L CaCl_2_ at 37°C for 4 hours for digestion. The supernatants were then centrifuged for 10 minutes at 200 *g*. Next, cells were resuspended in low‐sugar Dulbecco's modified Eagle's medium (DMEM, 1 g/L; Gibco, Life Technologies, Mulgrave, Vic, Australia) with 10% foetal bovine serum (FBS; Pan‐Biotech GmbH, Aidenbach, Germany) and cultured under standard conditions (humidified atmosphere, 37°C, 5% CO_2_). Medium was refreshed every 3 days to eliminate non‐adherent cells. Adherent cells were cultured further until the third passage. Freshly isolated UC‐MSCs were then prepared for further experiments. To evaluate their potential for osteogenic and adipogenic differentiation, UC‐MSCs were cultured with specific osteogenesis differentiation medium or specific adipogenesis differentiation medium (Gibco, Life Technologies, Carlsbad, CA); after 21 days of incubation, the samples were stained with Alizarin Red S or Oil Red O, respectively. A colony‐forming unit assay was performed as previously described.^29^ After 12 days of incubation, cells were then detected by staining with a 0.5% crystal violet solution.

### 2.2 Flow cytometry

Flow cytometry was used to identify the characteristics of the cultured cells. Cell surface antigens on the UC‐MSCs, including CD105‐FITC, CD90‐APC, CD73‐FITC, CD44‐APC, CD34‐PE‐Cy7, CD166‐PE, CD29‐PE, CD45‐PE‐Cy7 and CXCR4‐APC, were evaluated as previously described.^30^ All antibodies were purchased from Becton Dickinson (San Diego, CA) and diluted to 1:500.

### 2.3 Experimental groups

Umbilical cord‐derived mesenchymal stem cells were divided into a 3‐methyladenine (3‐MA) group (10 mmol/L 3‐MA; Sigma‐Aldrich), a rapamycin group (3 mmol/L rapamycin; Sigma‐Aldrich) and a control group (common UC‐MSC medium consisting of low glucose DMEM with 10% FBS; 27964826, 24730420) and incubated at 37°C for 24 hours. Solutions of 3‐MA and rapamycin were prepared as described in a previous study.^27^

### 2.4 Cytotoxicity assay

Umbilical cord‐derived mesenchymal stem cells were seeded in 96‐well plates at a density of 2000 cells/well. After treatment for 12, 24 and 48 hours, a Cell Counting Kit‐8 (CCK‐8; Dojindo, Kumamoto, Japan) was used according to the manufacturer's instructions. An Annexin V‐propidium iodide apoptosis detection kit (Kaiji Bio‐Technology Co. Ltd., Nanjing, China) was used to quantify apoptosis of UC‐MSCs according to the manufacturer's protocol. In brief, UC‐MSCs were seeded in 6‐well plates at a density of 5 × 10^5^ cells/well. When the cells had reached a confluency of 70%‐80%, they were treated with 3‐MA or rapamycin for 24 hours. The cells were then collected and stained with Annexin V‐propidium iodide for 15 minutes. The apoptosis rate was analysed using flow cytometry. Next, the UC‐MSCs were seeded in 6‐well plates at a density of 5 × 10^5^ cells/well. After exposure to the different treatments for 0, 24, 48, 72 and 96 hours, the UC‐MSCs were collected, stained with 0.4% trypan blue and counted in a hemocytometer (Neubauer Improved Bright Line Hemacytometer, Marien Feld, Germany).

### 2.5 GFP‐LC3B plasmid transient transfection

UC‐MSCs were transfected with the GFP‐LC3B plasmid using Lipofectamine 3000 (Invitrogen, Carlsbad, CA) according to the manufacturer's protocol. After 24 hours of incubation, cells were exposed to the different treatments for another 24 hours and then visualized under a fluorescence microscope.

### 2.6 Transmission electron microscopy

Umbilical cord‐derived mesenchymal stem cells were collected and fixed in 2.5% glutaraldehyde at 4°C overnight. The samples were then cut into ultrathin sections (80 nm) and stained with uranyl acetate and lead citrate. Autophagosomes in the UC‐MSCs were observed under an HT7700 transmission election microscope (Hitachi High‐Technologies Corp., Tokyo, Japan).

### 2.7 siRNA transfection

Umbilical cord‐derived mesenchymal stem cells were transfected with human siRNA to knock down CXCR4 expression using Lipofectamine RNAiMAX (Life Technologies) and a synthetic CXCR4 siRNA sequence (5ꞌ‐GGTGGTCTATGTTGGCGTCTG‐3ꞌ) according to the manufacturer's instructions.

### 2.8 Western blotting

Protein expression levels of LC3B, Beclin1 and CXCR4 in the UC‐MSCs and of CXCL12 (Cell Signaling Technology, Danvers, MA) in the liver tissues were detected by Western blot assays. Total protein was extracted from the cells and tissues using lysis buffer (Kaiji). LC3B, Beclin1, CXCR4, CXCL12 and GAPDH were measured and analysed as previously described.^7^ The following first antibodies were used: LC3B‐, Beclin1, CXCR4, CXCL12, and GAPDH (1:1000; from Cell Signalling Technology), and the secondary antibody was used: (anti‐rabbit IgG) (1:5000; Sigma‐Aldrich).

### 2.9 Real‐time reverse transcriptase‐polymerase chain reaction

Specific primers for all genes used in the study are listed in Table S1. After extraction of total RNA from the UC‐MSCs and liver tissue using TRIzol reagent (Invitrogen), complementary DNA was synthesized using a reverse transcription kit (Roche Applied Science, Mannheim, Germany) following the manufacturer's instructions. Reverse transcription‐polymerase chain reaction was performed using a QuantiTect SYBR Green PCR kit (Roche Applied Science) and analysed using a LightCycler 480 real‐time PCR system (Roche Diagnostics, Indianapolis, IN).

### 2.10 Scratch migration assay

The scratch migration assay was performed using 6‐well plates (Corning Costar, Corning, NY) at a density of 3 × 10^6^ cells/well. When the cultured UC‐MSCs had reached 70%‐80% confluence, a line was scratched using a 1‐mL pipette tip. The cells were then randomly divided into six groups and treated with 3‐MA, rapamycin, rapamycin + AMD3100, a CXCR4 inhibitor (5 mg/mL; Cayman Chemical Company, Ann Arbor, MI), rapamycin + CXCL12, a CXCR4 agonist (50 ng/mL; R&D Systems, Minneapolis, MN), shCXCR4‐UC‐MSCs + rapamycin or common UC‐MSC medium. Each well was also treated with mitomycin C (10 µg/mL; Sigma‐Aldrich) to inhibit cell proliferation. Images were acquired at 0 and 24 hours under an inverted microscope (Leica, Mannheim, Germany) for cell counting.

### 2.11 Transwell experiment

The Transwell assay was used to determine the migratory ability of UC‐MSCs in 24‐well plates containing an 8‐µm pore membrane (Corning Costar). Control UC‐MSCs were cultured in DMEM without FBS in the upper chamber at a density of 1 × 10^5^ cells/well; DMEM with 10% FBS was added to the bottom chamber. The other five groups were supplemented with 3‐MA, rapamycin, rapamycin + AMD3100, rapamycin + CXCL12, or siCXCR4‐UC‐MSCs + rapamycin and cultured at 5% CO_2_ and 37°C for 24 hours. Subsequently, a wet cotton swab was used to remove the UC‐MSCs from the upper chamber which did not migrate, and trypan blue was applied to stain the cells which had migrated. The UC‐MSCs that migrated to the bottom of the chamber were observed and imaged using a light microscope (×200, Leica). The transferred cells in each group were counted in five randomly selected fields of the Matrigel.

### 2.12 Enzyme‐linked immunosorbent assay

Umbilical cord‐derived mesenchymal stem cells were seeded in 6‐well plates at a density of 5 × 10^5^ cells/well. After the cells reached 70%‐80% confluence, they were exposed to the different study treatments according to group allocation. Supernatants were then collected after 24 hours treatment, and the levels of cytokines secreted, including interleukin (IL)‐10, prostaglandin E2, indoleamine‐2, 3‐dioxygenase (IDO) and transforming growth factor (TGF)‐β1, were determined using human enzyme‐linked immunosorbent assays (ELISA; EIAab, Wuhan, China) according to the manufacturer's protocols.

### 2.13 Creation of the liver I/R model and transplantation of UC‐MSCs

All the animal experiments performed were approved by the Animal Care and Use Committee of Sun Yat‐sen University. The mouse liver I/R model was induced as previously described.^31^ All C57BL/6 mice (male, aged 8‐10 weeks) received an intraperitoneal injection of 0.6% pentobarbital sodium (100 µL/10 g) for anaesthesia. Blood flow to 70% of the liver was occluded for 90 minutes with an atraumatic vascular clamp. After the liver I/R model was established, the mice were randomly divided into five groups (6‐7 mice per group) to receive PBS (100 µL), naive UC‐MSCs (1 × 10^6^ cells/100 µL per mouse), rapamycin‐UC‐MSCs, rapamycin‐UC‐MSCs + AMD3100 (5 mg/mL) or 3‐MA (30 mg/kg) + UC‐MSCs via a peripheral vein after reperfusion. All the mice were sacrificed after 24 hours of reperfusion. In the sham group, only a midline laparotomy incision was made.

CellTracker™ Green CMFDA (Molecular Probes, Invitrogen Carlsbad, CA) was used to label and track the UC‐MSCs according to the manufacturer's protocol and created four additional groups. Thin 6‐µm frozen sections of lung and liver tissues were prepared and the number of CellTracker‐labelled cells was counted to monitor the engraftment of UC‐MSCs.

### 2.14 Liver injury assay

Serum alanine and aspartate aminotransferase levels were measured using a 7180 Biochemical Analyzer (Hitachi). After preparation of paraffin‐embedded liver tissue sections measuring 4 µm in thickness and stained with haematoxylin and eosin, the histological characteristics of the liver tissues were scored according to Suzuki's criteria (Table S2).^32^

### 2.15 Immunohistochemistry

Liver tissue sections 4 µm in thickness were dewaxed, rehydrated and prepared for immunohistochemical staining. Expression of caspase 3 and Ly6G protein (Abcam, Cambridge, MA) in the liver tissue specimens was evaluated after incubation with the relevant primary antibodies. Caspase 3 and Ly6G in the liver sections were measured as previously described.^33^ The sections were assessed under a light microscopy (×200). Infiltration of neutrophils was quantified by counting the number of Ly6G^+^ cells in eight randomly selected regions in two sections per mouse. And the percentage of caspase 3+cells was calculated as the number of caspase 3+cells/total number of nuclei.

### 2.16 Assessment of ROS production in liver tissues

An OxiSelect intracellular ROS assay kit was used to measure generation of ROS and an ELISA kit was used to detect 8‐isoprostane levels (both kits from Cayman Chemical Company) according to the manufacturers’ instructions to evaluate oxidative stress in the liver tissues.

### 2.17 Statistical analysis

Data are presented as the mean ± standard deviation. The statistical significance of the data was determined by one‐way analysis of variance or the Student's *t* test, as appropriate. A *P*‐value <0.05 was considered statistically significant.

## 3 RESULTS

### 3.1 Characterization of UC‐MSCs

As shown in Figure S1A, CD105, CD44, CD29, CD90, CD73 and CD166 were expressed on the cell surface but CD45 and CD34 were not. The cells obtained showed the capacity to form colonies (Figure S1B) and undergo adipogenic and osteogenic differentiation (Figure S1C).

### 3.2 Autophagy in UC‐MSCs is enhanced by rapamycin and decreased by 3‐MA

**Figure 1:**
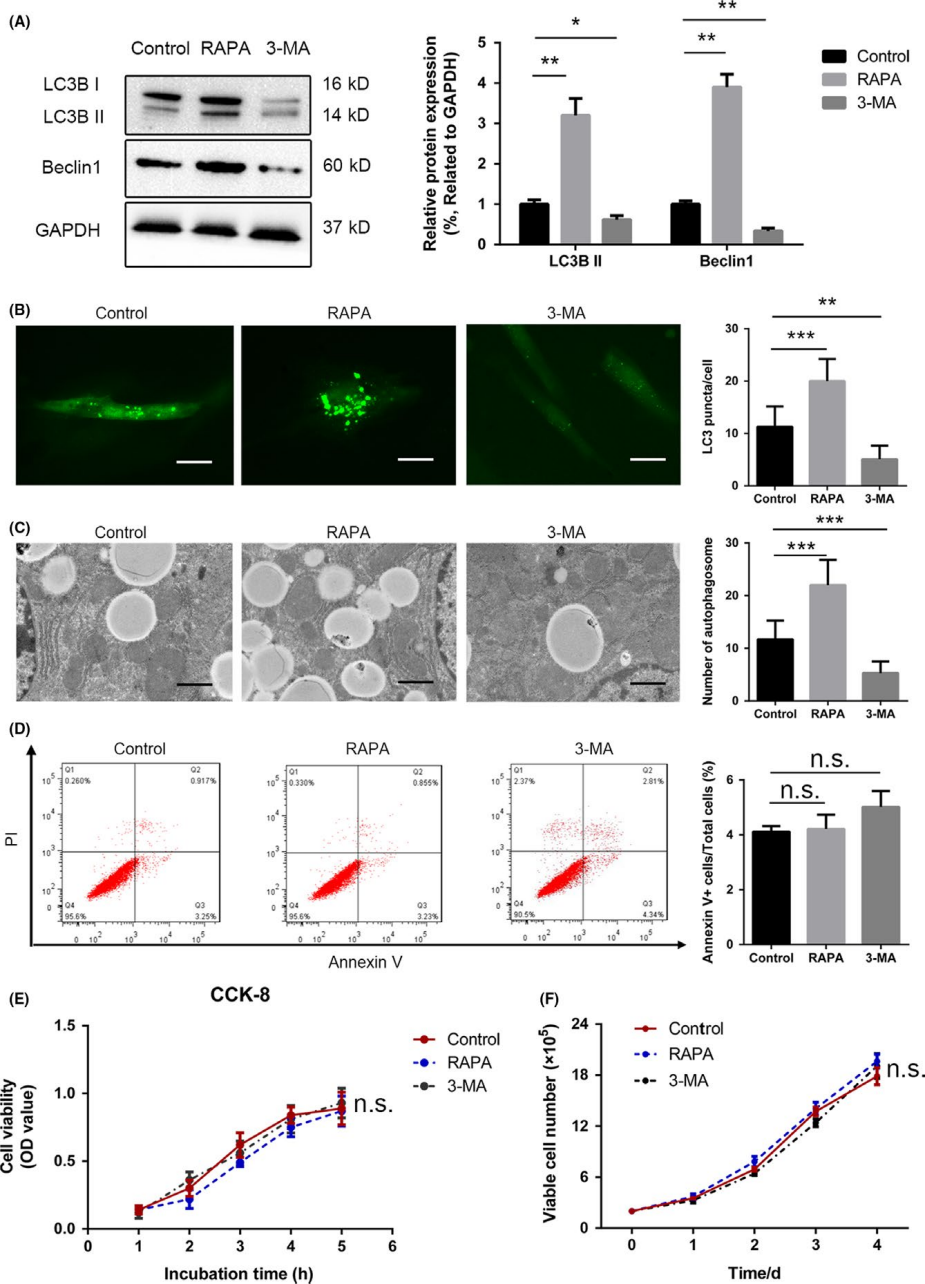
Regulation of autophagy in UC‐MSCs. A, UC‐MSCs were exposed to rapamycin or 3‐MA for 24 h and autophagy levels were determined by analysing LC3B II/I ratio and Beclin1/GAPDH with anti‐LC3B antibody and anti‐Beclin1 antibody. GAPDH housekeeping protein was used as control. Semi‐quantitative analysis of Western blot about Beclin1 protein expression and the change of LC3B II/I ratio. B, After 24 h of transfection with a lentiviral vector containing GFP‐LC3B, UC‐MSCs were subjected to various treatment conditions. Subsequently, puncta‐like staining was detected under on fluorescence microscopy (×200). Green puncta were counted from five cells in each group. C, Transmission electron microscopy was used to observe autophagosomes in UC‐MSCs in the different treatment groups. The number of autophagosomes was counted in representative images from three independent experiments. D, Apoptosis of UC‐MSCs was measured by Annexin V/propidium iodide staining after exposure to the different treatments. The results of the statistical analysis for the percentage of Annexin V‐positive neutrophils are shown. Data are presented as the mean ± standard error of the mean (n = 5) for each group. E, UC‐MSCs were subjected to a CCK‐8 assay after treatment with rapamycin or 3‐MA for 1, 2, 3, 4 and 5 h to assess cell viability. F, UC‐MSCs were counted after exposure to rapamycin or 3‐MA for 0, 1, 2, 3 and 4 d. Data for control and treated groups are presented as the mean ± standard error of the mean. *P < 0.05, **P < 0.01, ***P < 0.001 (all one‐way analysis of variance). 3‐MA, 3‐methyladenine; UC‐MSCs, umbilical cord‐derived mesenchymal stem cells

It is well known that rapamycin induces autophagy and that 3‐MA inhibits it. Western blotting revealed significant increases in the LC3B II/I and Beclin1/GAPDH ratios after the UC‐MSCs had been preconditioned with rapamycin for 24 hours, whereas preconditioning with 3‐MA had the opposite effect (Figure 1A). UC‐MSCs were also transfected with a lentiviral vector carrying GFP‐LC3B before treatment with various reagents. Significantly more green puncta were observed in the UC‐MSCs after exposure to rapamycin for 24 hours, whereas there was a decrease in the number of green puncta after exposure to 3‐MA for 24 hours (Figure 1B). In addition, transmission electron microscopy was performed to directly observe and count the number of autophagosomes. The number of autophagosomes formed increased markedly after preconditioning with rapamycin but decreased significantly after preconditioning with 3‐MA (Figure 1C) when compared with the control group. Finally, we found that the phenotype, multi‐differentiation ability and proliferation capacity of UC‐MSCs after treatment with rapamycin did not exhibit significantly difference compared with the control group (Figure S1A‐C).

### 3.3 Changes in autophagy did not affect apoptosis or proliferation of UC‐MSCs but changed their immunoregulatory potential

The UC‐MSC apoptosis and proliferation rates were calculated after treatment with rapamycin or 3‐MA. According to the flow cytometry analysis, treatment with 3‐MA or rapamycin did not accelerate the rate of apoptosis (4.167% in the control group, 4.085% in the rapamycin group and 7.15% in the 3‐MA group, Figure 1D). Similarly, the CCK‐8 assay and cell counting method did not identify any significant difference in the cell proliferation rate between these three groups (Figure 1E,F). We also attempted to measure the immunomodulatory cytokines and factors released by UC‐MSCs after the various treatments. ELISA results demonstrated a positive association of TGF‐β1, IDO, IL‐10 and prostaglandin E2 levels with autophagy in UC‐MSCs. Specifically, the group treated with rapamycin had significantly elevated levels of IL‐10, TGF‐β1, IDO and prostaglandin E2 in the UC‐MSC culture supernatant compared to the control group (compared to the control group, increased 1.7‐, 2.4‐, 1.4‐ and 1.5‐fold, respectively), whereas 3‐MA blocked these effects (based on the control group, decreased 1.8‐, 1.3‐, 1.4‐ and 1.5‐fold, respectively) (Figure S2A). Consistent with the RT‐qPCR results, the mRNA expression levels of TGF‐β1, IDO and IL‐10 were increased by preconditioning with rapamycin (compared with the control group, the mRNA expression enhanced 2.4‐, 1.5‐ and 2‐fold, respectively) but decreased by preconditioning with 3‐MA (based on the control group, the mRNA expression reduced 1.4‐, 1.3‐ and 2‐fold, respectively) (Figure S2B).

### 3.4 UC‐MSC migration in vitro is enhanced by preconditioning with rapamycin but inhibited by preconditioning with 3‐MA

**Figure 2:**
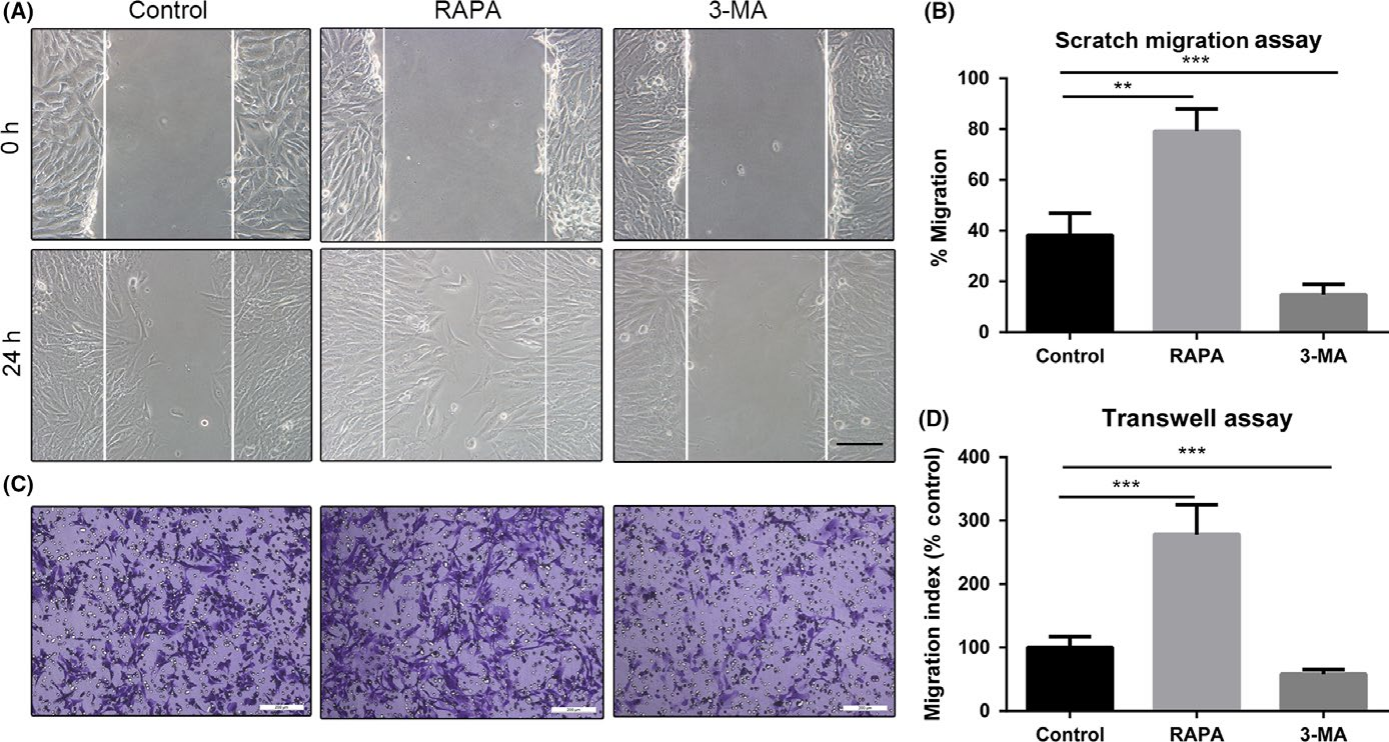
Scratch migration and Transwell assays showing that a change in autophagy alters the migration of UC‐MSCs. A, Representative images of cell migration in a scratch migration assay (100×). B, Results of statistical analysis of the number of migrated cells are shown. Data are presented as the mean ± standard error of the mean. C, Representative images of cell migration in a Transwell system. Scale bar: 200 µm. D, Results of statistical analysis of the number of migrated cells are shown. Data are presented as the mean ± standard error of the mean. **P < 0.01 and ***P < 0.001 (both by one‐way analysis of variance). UC‐MSCs, umbilical cord‐derived mesenchymal stem cells

Scratch migration and Transwell assays were used to examine the effect of preconditioning with rapamycin and with 3‐MA on migration of UC‐MSCs in vitro. The results of the scratch migration assay showed that the migratory ability of UC‐MSCs was increased by 24 hours of preconditioning with rapamycin but inhibited by 24 hours of preconditioning with 3‐MA (Figure 2A). The results of the Transwell assays were similar (based on the control group, increased 2.8‐fold in the rapamycin group, and decreased 1.7‐fold in the 3‐MA group; Figure 2B).

### 3.5 Autophagy‐enhanced UC‐MSC migration depends on the CXCR4/CXCL12 axis in vitro

**Figure 3:**
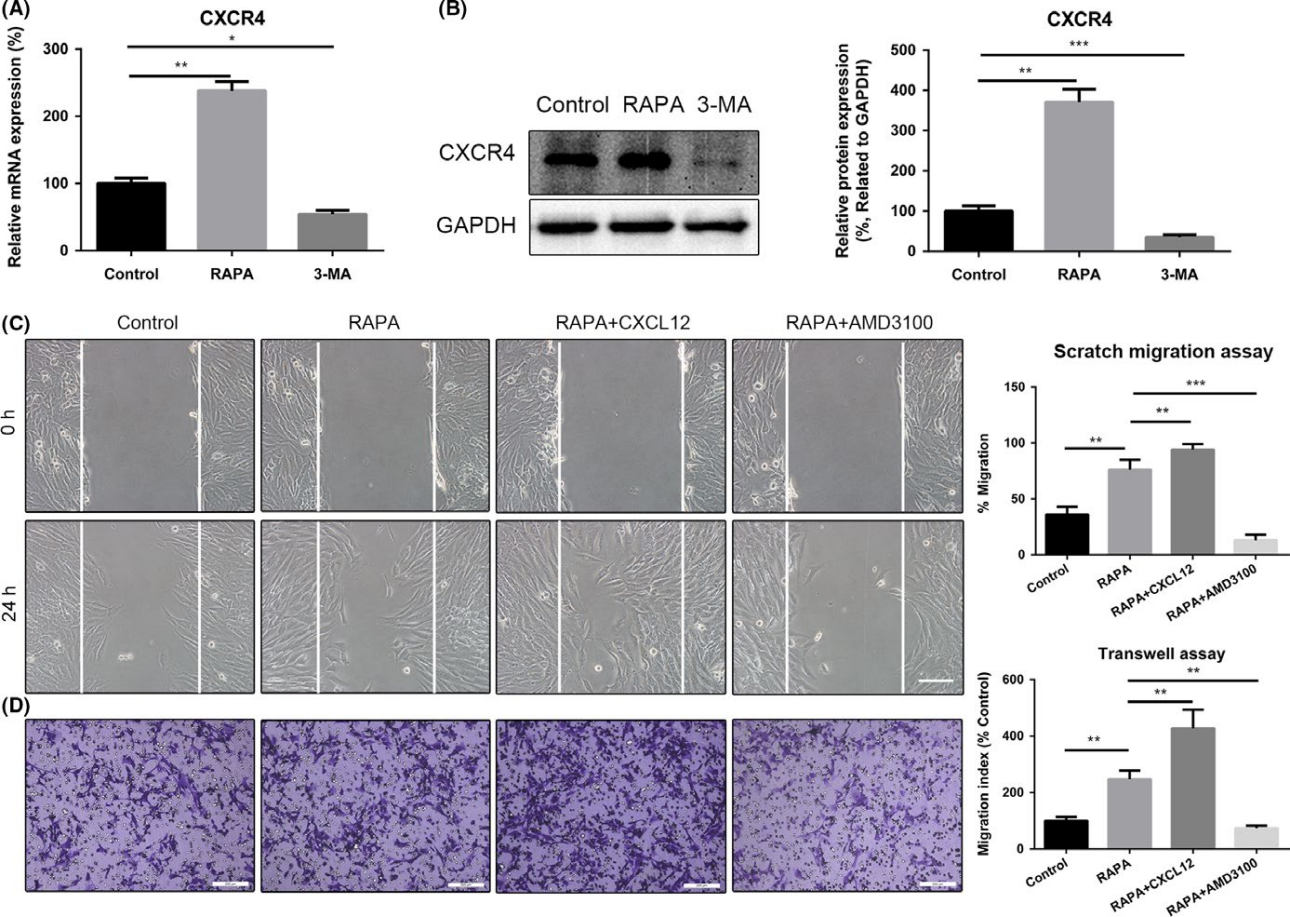
Pre‐treatment with rapamycin enhances migration of UC‐MSCs by upregulating expression of CXCR4. A, Relative mRNA expression of CXCR4 was determined by real‐time polymerase chain reaction. B, Expression of CXCR4 protein was detected by Western blotting assays. Results of statistical analysis of relative density of CXCR4 are shown. C, Representative images of migration of UC‐MSCs in a scratch migration assay after addition of rapamycin, rapamycin +CXCL12 or rapamycin +AMD3100 (×100). Scale bar: 200 µm. Results of statistical analysis of the number of migrated cells are shown. Data are presented as the mean ± standard error of the mean. D, Representative images of migration of UC‐MSCs in a Transwell system after addition of rapamycin, rapamycin +CXCL12 or rapamycin +AMD3100. Scale bar: 200 µm. Results of statistical analysis of the number of migrated cells are shown. Data are presented as the mean ± standard error of the mean. *P < 0.05, **P < 0.01, ***P < 0.001 (all one‐way analysis of variance). UC‐MSCs, umbilical cord‐derived mesenchymal stem cells

CXCR4 mRNA and protein expression levels in UC‐MSCs were assessed by using RT‐qPCR and Western blot analysis, respectively. Preconditioning with rapamycin for 24 hours markedly increased CXCR4 expression in UC‐MSCs, whereas 3‐MA reduced this expression (Figure 3A,B). Similar results were obtained for the surface expression levels of CXCR4 on UC‐MSCs obtained by the flow cytometry assay (Figure S3A). Scratch migration assay results showed that addition of CXCL12 significantly enhanced the ability of rapamycin to boost the migratory capacity of UC‐MSCs (Figure 3C); however, AMD3100 markedly blocked this effect (Figure 3C). The Transwell assay results confirmed these phenomena (compared with the rapamycin group, added with CXCL12 increased 1.7‐fold UC‐MSCs migration, whereas treated along with AMD3100 reduced 1.4‐fold cells migration) (Figure 3D). In addition, to further confirm the involvement of CXCR4, we transfected siRNA‐mediated CXCR4 (siCXCR4) into UC‐MSCs and found that transfection of siCXCR4 led to suppress not only mRNA expression, but also protein expression of CXCR4 in UC‐MSCs (Figure S4A,B). Concomitantly, the effect of rapamycin on enhancing UC‐MSCs migration was remarkably reversed (Figure S4C,D).

### 3.6 Transfusion of rapamycin‐preconditioned UC‐MSCs improves recovery of hepatic function and attenuates pathological changes in the liver

**Figure 4:**
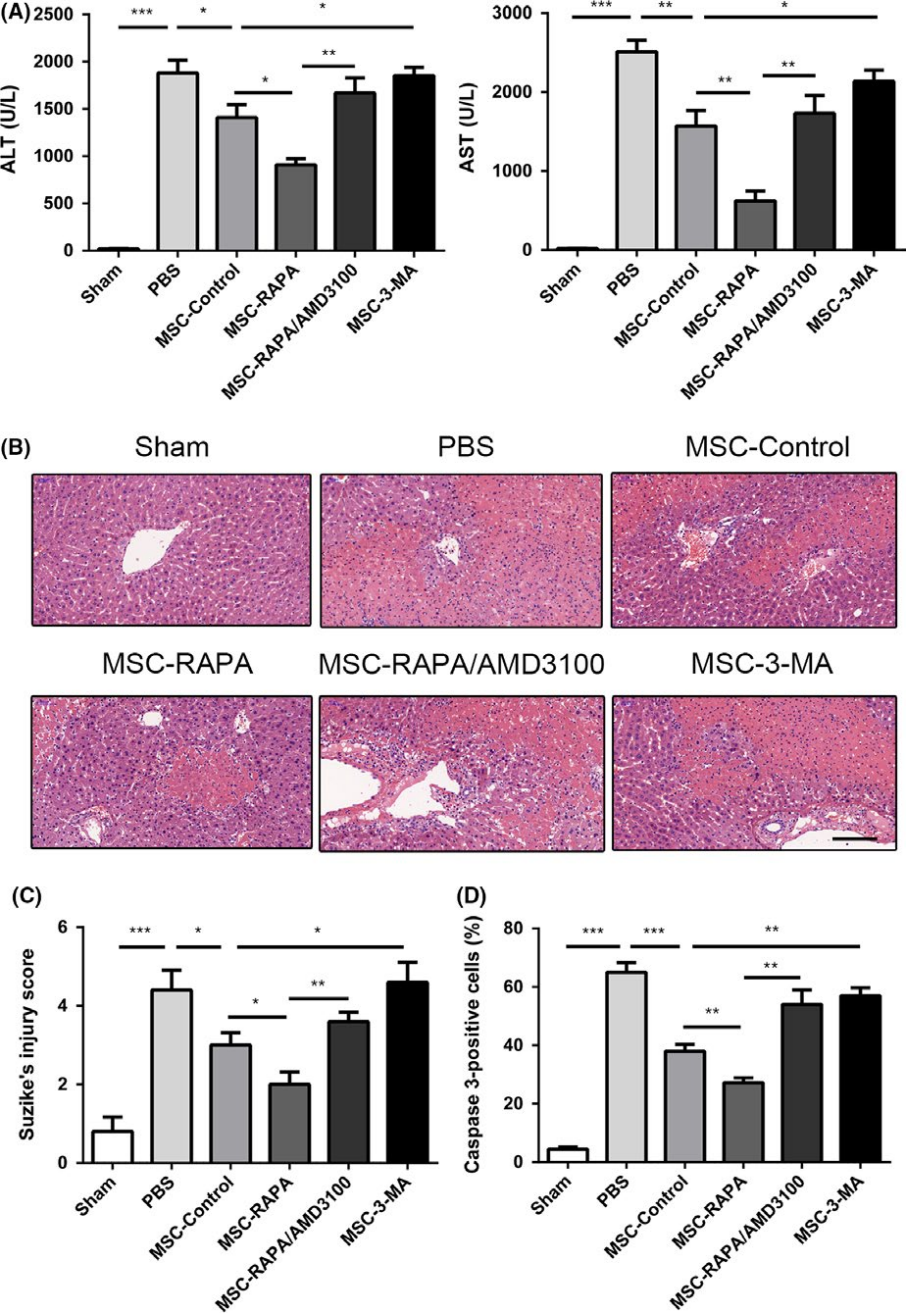
Enhancement of autophagy in UC‐MSCs is shown to protect the liver by decreasing levels of serum biomarkers and the histological features of hepatic injury after ischaemia/reperfusion (I/R) injury in vivo. A, Serum alanine and aspartate aminotransferase levels were detected after I/R injury in each treatment group. Data are shown as the mean ± standard error of the mean (n = 6 mice/group). B, Haematoxylin and eosin staining of liver tissues in each group to assess the amount of liver damage after I/R injury. Scale bar: 200 µm. C, Suzuki's injury score for each group calculated by randomly selecting five fields in each tissue sample. Results of statistical analysis are presented as the mean ± standard error of the mean (n = 6 mice/group). D, Amount of caspase‐3 in each group was assessed using immunohistochemistry to determine the percentage of caspase‐3‐positive cells in the liver. Statistical analysis was performed to determine the number of caspase‐3‐positive cells. Data are shown as the mean ± standard error of the mean (n = 6 mice/group). *P < 0.05, **P < 0.01, ***P < 0.001 (one‐way analysis of variance). UC‐MSCs, umbilical cord‐derived mesenchymal stem cells

We evaluated the amount of liver injury in each group to determine whether preconditioning of UC‐MSCs with rapamycin improves hepatic performance after I/R injury. As shown in Figure 4A, UC‐MSCs preconditioned with rapamycin significantly reduced the alanine and aspartate aminotransferase levels. However, preconditioning with 3‐MA markedly decreased the ability of UC‐MSCs to protect the liver. Furthermore, staining of liver sections with haematoxylin and eosin showed that administration of rapamycin‐preconditioned UC‐MSCs significantly inhibited apoptosis of cells and destruction of the hepatic lobules, in addition to reducing the Suzuki score (Figure 4B,C); 3‐MA also weakened these effects of UC‐MSCs (Figure 4B,C). Furthermore, cleaved caspase‐3‐positive cells were counted to confirm further the effects of autophagy on UC‐MSCs (Figure 4D). As shown in Figure 4D, we found that UC‐MSCs could significantly reduce the percentage of caspase‐3‐positive cells in the liver after I/R injury, and rapamycin exhibited an important role in promoting this effect of UC‐MSCs. However, all these beneficial effects of rapamycin preconditioning were reversed by AMD3100.

### 3.7 Transfusion of rapamycin‐preconditioned UC‐MSCs attenuates the inflammatory response and reduces oxidative stress in the liver after I/R injury

**Figure 5:**
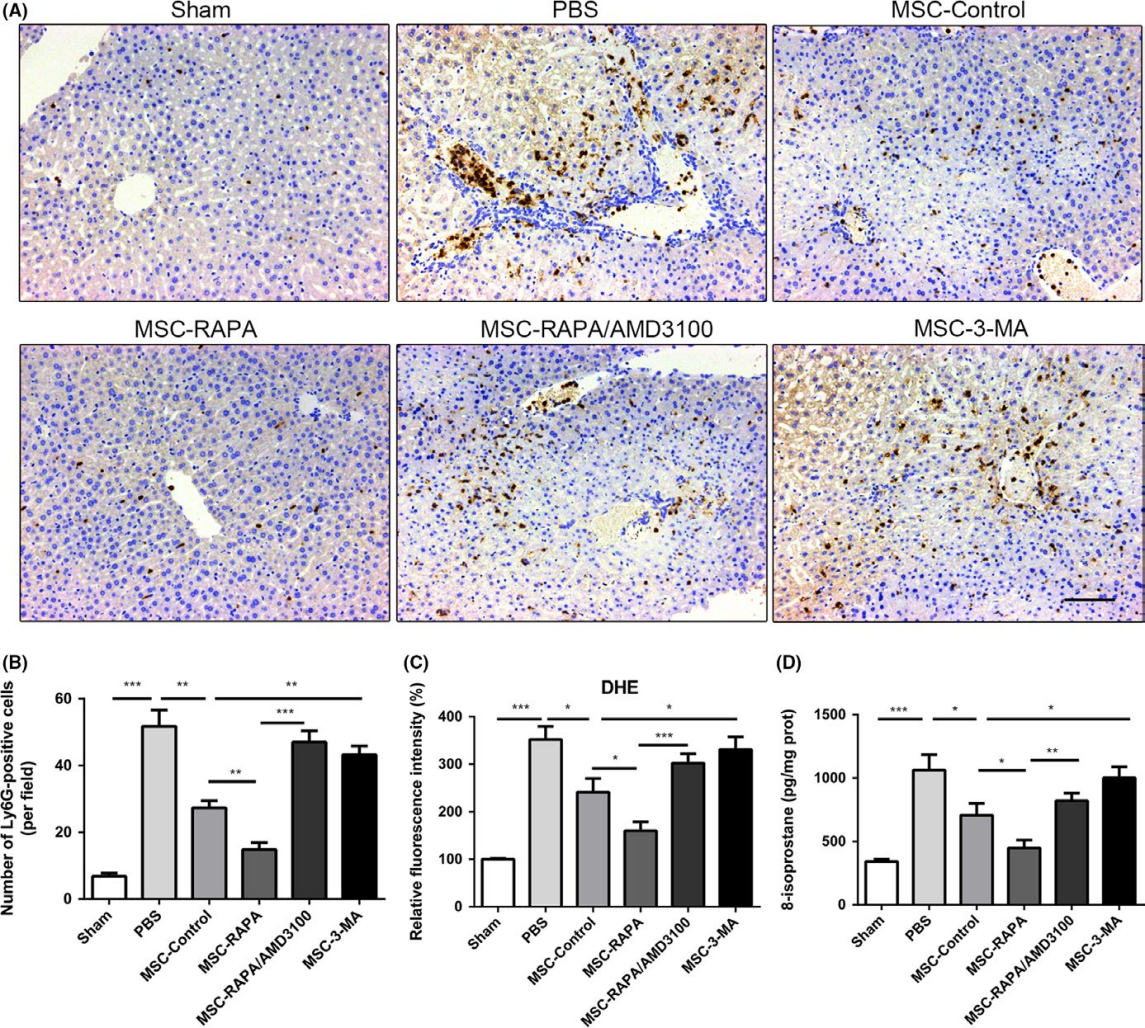
Induction of autophagy strengthened the ability of UC‐MSCs to inhibit infiltration of neutrophils into the liver and hepatic oxidative stress after ischaemia/reperfusion injury. A, Ly6G is a biomarker of neutrophils. Representative images of Ly6G‐positive cells were observed in each group by immunohistochemistry. Scale bar: 200 µm. B, The percentage of Ly6G‐positive cells were measured, and data are shown as the mean ± standard error of the mean (n = 7 mice/group). C, Fluorescence intensity of dehydroergosterol was measured to determine the levels of reactive oxygen species in liver samples. Data are shown as the mean ± standard error of the mean (n = 7 mice/group). D, 8‐isoprostane levels in each group were detected by enzyme‐linked immunosorbent assay. Data are shown as the mean ± standard error of the mean (n = 7 mice/group). *P < 0.05, **P < 0.01, ***P < 0.001 (all by analysis of variance). UC‐MSCs, umbilical cord‐derived mesenchymal stem cells

There is compelling evidence that infiltration of neutrophils plays an important role in the pathophysiological changes that occur in the liver after I/R injury. Therefore, we counted the number of neutrophils, that is, Ly6G‐positive cells, in the liver specimens from each group. Compared with the control UC‐MSCs, the rapamycin‐preconditioned UC‐MSCs were better able to prevent infiltration of neutrophils into the liver tissues. However, treatment with AMD3100 significantly reversed this effect. Furthermore, pre‐treatment with 3‐MA weakened the ability of UC‐MSCs to inhibit neutrophil infiltration in the liver specimens (Figure 5A,B). To investigate the inflammatory microenvironment in the liver, we further evaluated the mRNA expression levels for cytokines, including IL‐1β, IL‐6 and tumour necrosis factor alpha, in the liver tissues. mRNA levels of all three cytokines were significantly decreased in the rapamycin‐preconditioned UC‐MSC group when compared with those in the UC‐MSC treatment group and the 3‐MA‐preconditioned UC‐MSC group (Figure S5). Similarly, added with AMD3100 inhibited rapamycin‐preconditioned UC‐MSCs to migrate to the damaged sites via blocking CXCL12/CXCR4 axis which influenced the anti‐inflammatory effects of UC‐MSCs.

One of the most important pathological features in liver I/R injury is overproduction of ROS, which results in oxidative stress. Therefore, we examined the effect of UC‐MSCs on the oxidative status of the liver samples after I/R injury in the different pre‐treatment groups. The results indicated a significant increase in oxidative stress in the liver samples after I/R injury. In addition, compared with the control group and the 3‐MA‐preconditioned UC‐MSC group, the group that received rapamycin‐preconditioned UC‐MSCs had the greatest reversal of these phenomena, with decreased production of ROS (Figure 5C) and significantly increased formation of 8‐isoprostane (Figure 5D). Furthermore, AMD3100 could block the effect of rapamycin on UC‐MSCs.

### 3.8 Preconditioning with rapamycin enhances UC‐MSC homing to the ischaemic liver via the CXCR4/CXCL12 axis

**Figure 6:**
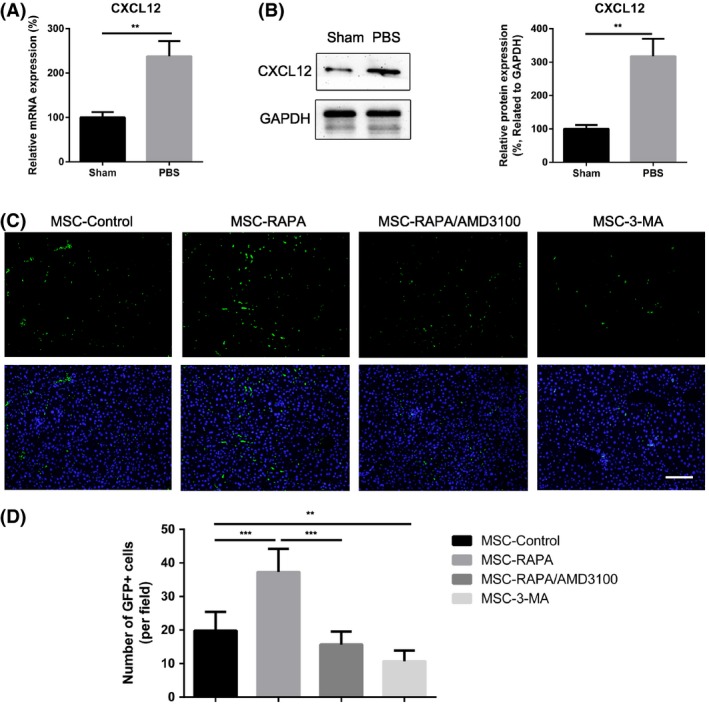
Induction of autophagy strengthened homing of UC‐MSCs to the liver after ischaemia/reperfusion injury via the CXCR4/CXCL12 axis. A, mRNA expression of CXCL12 in liver tissues was detected using real‐time polymerase chain reaction. Data are shown as the mean ± standard error of the mean (n = 7 mice/group). B, Protein expression of CXCL12 in liver tissues was evaluated using Western blotting assays. Data are shown as the mean ± standard error of the mean (n = 7 mice/group). Semi‐quantitative analysis of Western blot about CXCL12 protein expression. C, CellTrackerTM Green CMFDA (green) labelled UC‐MSCs were used to examine the count of UC‐MSCs engraftment in the liver tissues from each group. Scale bar: 200 µm. D, Quantification of migrated UC‐MSCs. Data are shown as the mean ± standard error of the mean (n = 7 mice/group). *P < 0.05, **P < 0.01, ***P < 0.001 (all by analysis of variance). UC‐MSCs, umbilical cord‐derived mesenchymal stem cells

To systematically evaluate the levels of chemokines in the damaged liver after I/R injury, we found that compared with the sham group, the group with damaged livers had significantly higher mRNA levels of CCL2, CCL3, CCL7, CXCL1, CXCL2, CXCL3 CXCL5, CXCL10 and CXCL12 (Figure 6A and Figure S5). We performed Western blotting analysis to measure the protein expression of CXCL12 in the liver samples for each group. As shown in Figure 6B, we found that the protein level of CXCL12 was significantly increased in the liver after I/R injury. Subsequently, the number of CellTracker Green CMFDA‐labelled UC‐MSCs was calculated in the lungs and ischaemic regions of the liver samples to determine the homing efficiency of UC‐MSCs. Compared with UC‐MSCs that did not receive any pre‐treatment, UC‐MSCs with rapamycin preconditioning had greater homing ability, whereas co‐preconditioning with AMD3100 significantly blocked the effect of rapamycin. In addition, 3‐MA preconditioning weakened the migratory capacity of UC‐MSCs towards ischaemic regions (Figure 6C,D). Furthermore, as shown in Figure S7, we found that induction of autophagy reduced the number of UC‐MSCs that were sequestered in the lung, whereas 3‐MA had the reverse effect and increased the number of UC‐MSCs in the lung. Overall, these results demonstrate that preconditioning with rapamycin enhances homing of UC‐MSCs to the ischaemic liver and reduces their retention in the lung via the CXCR4/CXCL12 axis.

## 4 DISCUSSION

A high incidence of liver I/R injury is closely associated with increased morbidity and mortality in patients undergoing major liver resection or liver transplantation. There is compelling evidence to suggest that the ability of MSCs to secrete multiple factors and differentiate into tissue‐specific cells directly can provide the basis for promising regenerative and immunosuppressive strategies that can be used in the repair of damaged tissues and inflammation‐related diseases.^34^, ^35^ Previous studies by our group and others have demonstrated the beneficial effects of MSCs in clinical trials and in rodent models of liver disease, including liver transplantation,^36^, ^37^ acute liver failure^4^ and liver I/R injury.^7^, ^38^

Although the therapeutic potential of MSCs in liver I/R injury is widely acknowledged, there are still several obstacles, the biggest being the low number of viable MSCs that migrate towards damaged tissues, especially after peripheral venous transfusion.^10^, ^39^ Various methods for manipulation of MSCs have been explored to rescue the migratory capacity and ability of these cells to home to specific sites of damage. Pre‐treatment with different cytokines or factors is generally accepted and has a small influence on the cell status. However, whether induction of by rapamycin would affect the migratory capacity of MSCs is still unknown. Autophagy is an evolutionarily conserved self‐degradation process and is traditionally considered to have an important role in maintaining the functions of the cell, removing abnormal cell proteins and organelles, and resisting starvation.^40^ Many studies have investigated the effects of autophagy on other essential functions of MSC, including their capacity for immunoregulation, vascularization and osteogenic differentiation.^21^, ^27^, ^41^, ^42^ Previous studies have also reported that autophagy affects the migratory ability of macrophages,^43^ cancer cells^44^ and arterial smooth muscle cells.^45^ However, the relationship between autophagy and migration of MSCs remains controversial. Yang et al^46^ found that SDF‐1α stimulated migration of dental pulp stem cells by activating autophagy, whereas Yeh^47^ showed that induction of autophagy by honokiol was negatively associated with migration of neuroblastoma cells. The current study is the first to detect that induction of autophagy in UC‐MSCs in the presence of rapamycin strengthened the migratory capacity of these cells, as evidenced by the results of a scratch migration assay and a Transwell assay. Moreover, 3‐MA, an autophagy inhibitor, significantly and substantially suppressed migration of UC‐MSCs. We also found that activation of autophagy not only promoted the roles of UC‐MSCs in liver I/R injury, but also enhanced the ability of UC‐MSCs to home towards the damaged liver tissue.

The CXCR4/CXCL12 axis participates in the migration of rapamycin‐strengthened MSCs. Given the evidence of high CXCL12 expression in damaged tissues,^13^, ^14^, ^15^ the interaction of CXCL12 with CXCR4 was demonstrated to mediate migration of MSCs in vitro^48^ and to regulate the homing of transfused cells towards specific tissues in vivo.^49^ Unfortunately, a small population of MSCs express CXCR4 during ex vivo expansion.^12^ Therefore, a variety of strategies have been used to upregulate CXCR4 expression in MSCs. Li suggested that pre‐treatment with tetramethylpyrazine enhanced homing of MSCs to the ischaemic brain in a rodent model of stroke by upregulating CXCR4.^50^ However, Li also reported that increased expression of CXCR4 in MSCs by ultrasound‐targeted microbubble destruction was positively associated with the number of MSCs that migrated to infarcted areas of myocardium.^51^ The effect of autophagy on expression of CXCR4 in cells is unclear. Singh et al showed that SIRT6 and hexokinase 2 activated autophagy and upregulated expression of CXCR4 in monocytes.^52^ However, stimulation of chemotactic G protein‐coupled receptors enhanced expression of CXCR4 in glioblastoma cells by suppressing formation of autophagosomes.^53^ In the present study, we first demonstrated that induction of autophagy by rapamycin enhanced the expression of CXCR4 on UC‐MSCs, as indicated by upregulation of the mRNA and protein expression of CXCR4 in UC‐MSCs, whereas 3‐MA significantly blocked these changes. These observations were confirmed by our finding that both AMD3100 and siCXCR4 weakened the ability of rapamycin to promote migration of UC‐MSCs and decreased the ability of these cells to protect the liver. Little is known about the molecular mechanism by which autophagy regulates migration of MSCs. Kubic et al showed that PAX3 and FOXD3 enhanced migration of melanoma cells by promoting expression of CXCR4,^54^ and Li et al reported that missing‐in‐metastasis protein downregulated CXCR4 expression in HeLa cells via Rab5, a small GTPase.^55^ Furthermore, PPAR gamma and p53 have been found to negatively regulate expression of the CXCR4 gene in breast cancer cells.^56^, ^57^ Moreover, several studies have reported that some lncRNAs and microRNAs affect expression of CXCR4 on cells.^58^, ^59^, ^60^ In addition, autophagy proteins were shown to upregulate phosphorylation of ERK and to activate the ERK‐related signalling pathway,^61^, ^62^ and the ERK pathway has been found to play an important role in promoting expression of CXCR4.^63^ The results of the present study indicate that induction of autophagy by rapamycin is the potential mechanism for the upregulated cell expression of CXCR4.

In conclusion, this study found that induction of autophagy by preconditioning with rapamycin can be used to strengthen the homing and migratory capacity of UC‐MSCs and to improve hepatic function after I/R injury. It is well known that the interaction between CXCL12 and CXCR4 may contribute to the chemotaxis of transfused UC‐MSCs. Our finding of increased CXCL12 expression in liver tissue after I/R injury indicates that preconditioning with rapamycin enhances the ability of UC‐MSCs to home towards ischaemic liver tissue by increasing the expression of CXCR4. Therefore, pre‐treatment with rapamycin may be a promising strategy to strengthen the therapeutic potential of UC‐MSCs in the treatment of liver I/R injury in the clinical setting.

## CONFLICT OF INTEREST

The authors declared no conflict of interest in this manuscript.
