# Supplementary material for: Preconditioning of umbilical cord‐derived mesenchymal stem cells by rapamycin increases cell migration and ameliorates liver ischaemia/reperfusion injury in mice via the CXCR4/CXCL12 axis
Source: Cell Prolif. 2018 Dec 10;52(2):e12546. doi: 10.1111/cpr.12546 (PMC6496237; doi:10.1111/cpr.12546)
Supplement: Supplementary file 1 [file CPR-52-e12546-s001.docx]

**SUPPLEMENTAL INFORMATION**

**Preconditioning of Umbilical Cord-Derived Mesenchymal Stem Cells by Rapamycin Increases Cell Migration and Ameliorates Liver Ischemia/Reperfusion Injury in Mice via the CXCR4/CXCL12 Axis**

**-SUPPLEMENTAL TABLE 1-2**

**-SUPPLEMENTAL FIGURES 1-7**

**Supplemental table 1. Primer sequences for real-time PCR**

| Gene symbol | Sequence direction | Sequence |
| --- | --- | --- |
| Human-CXCR4 | **Forward primer** | **5’- TCATCCTCATCCTGGCTTTC -3’** |
|  | **Reverse primer** | **5’- CAAACTCACACCCTTGCTTG -3’** |
| Mouse-CXCL12 | **Forward primer** | **5’- GAGCCAACGTCAAGCATCTG -3’** |
|  | **Reverse primer** | **5'- CAATGCACACTTGTCTGTTG -3’** |
| Human-IL-10 | **Forward primer** | **5’- TGTTGAGCCAGTCTCTGCTG -3’** |
|  | **Reverse primer** | **5’- GCATCACCTCCTCCAGGTAA -3’** |
| Human-IDO | **Forward primer** | **5’- GCCAGCTTCGAGAAAGAGTTG -3’** |
|  | **Reverse primer** | **5’- ATCCCAGAACTAGACGTGCAA -3’** |
| Human-TGF-β1 | **Forward primer** | **5’- CAATTCCTGGCGATACCTCAG -3’** |
|  | **Reverse primer** | **5’- GCACAACTCCGGTGACATCAA -3’** |
| Mouse-IL-1β | **Forward primer** | **5’- TGCAGCTGGAGAGTGTGGAT -3’** |
|  | **Reverse primer** | **5’- GACACGGATTCCATGGTGAA -3’** |
| Mouse-IL-6 | **Forward primer** | **5’- TCCATCCAGTTGCCTTCTTG -3’** |
|  | **Reverse primer** | **5’- CCACGATTTCCCAGAGAACA -3’** |
| Mouse-TNF-α | **Forward primer** | **5’- AGCACAGAAAGCATGATCCG -3’** |
|  | **Reverse primer** | **5’- CTGATGAGAGGGAGGCCATT -3’** |
| Mouse-CCL2 | **Forward primer** | **5’- TTAAAAACCTGGATCGGAACCAA -3’** |
|  | **Reverse primer** | **5’- GCATTAGCTTCAGATTTACGGGT -3’** |
| Mouse-CCL3 | **Forward primer** | **5’- GCTCAACATCATGAAGGTCTCC -3’** |
|  | **Reverse primer** | **5’- TGCCGGTTT CTCTTAGTCAGG -3’** |

| Gene symbol | Sequence direction | Sequence |
| --- | --- | --- |
| Mouse-CCL7 | **Forward primer** | **5’- TGTGCCTGCTGCTCATAGCC -3’** |
|  | **Reverse primer** | **5’- ACATAGCAGCATGTGGATGCATTG -3’** |
| Mouse-CCL19 | **Forward primer** | **5’- CCTGGGAACATCGTGAAAGC -3’** |
|  | **Reverse primer** | **5’- TAGTGTGGTGAACACAACAGC -3’** |
| Mouse-CXCL1 | **Forward primer** | **5’- TCGAGACCATTTACTGCAACAG -3’** |
|  | **Reverse primer** | **5’- CATTGCCGGTGGAAATTCCTT -3’** |
| Mouse-CXCL2 | **Forward primer** | **5’- CCAACCACCAGGCTACAGG -3’** |
|  | **Reverse primer** | **5’- GCGTCACACTCAAGCTCTG -3’** |
| Mouse-CXCL3 | **Forward primer** | **5’- GAAAGGAGGAAGCCCCTCAC -3’** |
|  | **Reverse primer** | **5’- TGGCCAGCCAAGGAATACTG -3’** |
| Mouse-CXCL4 | **Forward primer** | **5’- CTCATAGCCACCCTGAAGAATG -3’** |
|  | **Reverse primer** | **5’- AGGCAGCTGATACCTAACTCT -3’** |
| Mouse-CXCL5 | **Forward primer** | **5’- GTTCCATCTCGCCATTCATGC -3’** |
|  | **Reverse primer** | **5’- GCGGCTATGACTGAGGAAGG -3’** |
| Mouse-CXCL9 | **Forward primer** | **5’- GGAGTTCGAGGAACCCTAGTG -3’** |
|  | **Reverse primer** | **5’- GGGATTTGTAGTGGATCGTGC -3’** |
| Mouse-CXCL10 | **Forward primer** | **5’- CCAAGTGCTGCCGTCATTTTC -3’** |
|  | **Reverse primer** | **5’- GGCTCGCAGGGATGATTTCAA -3’** |
| Mouse-CXCL11 | **Forward primer** | **5’- CATTTTGACGGCTTTCATCC -3’** |
|  | **Reverse primer** | **5’- AAGGTCACAGCCATAGCCCT -3’** |

| Gene symbol | Sequence direction | Sequence |
| --- | --- | --- |
| Human-GAPDH | **Forward primer** | **5’- CTGACTTCAACAGCGACACC -3’** |
|  | **Reverse primer** | **5’- GTGGTCCAGGGGTCTTACTC -3’** |
| Mouse-β-actin | **Forward primer** | **5’- GTTGTCGACGACGAGCG -3’** |
|  | **Reverse primer** | **5’- GCACAGAGCCTCGCCTT -3’** |

**Supplemental table 2. The Suzuki’s criteria**

| Grade | Hyperaemia (%) | Necrosis (%) | Physalization (%) |
| --- | --- | --- | --- |
| 0 | 0 (None) | 0 (None) | 0 (None) |
| 1 | 10 (Minimal) | 10 (Minimal) | 10 (Minimal) |
| 2 | 11-30 (Mild) | 11-30 (Mild) | 11-30 (Mild) |
| 3 | 31-60 (Moderate) | 31-60 | 31-60 |
| 4 | >60 (Severe) | >60 (Severe) | >60 (Severe) |

**Supplemental figures and supplemental figure legends**

**
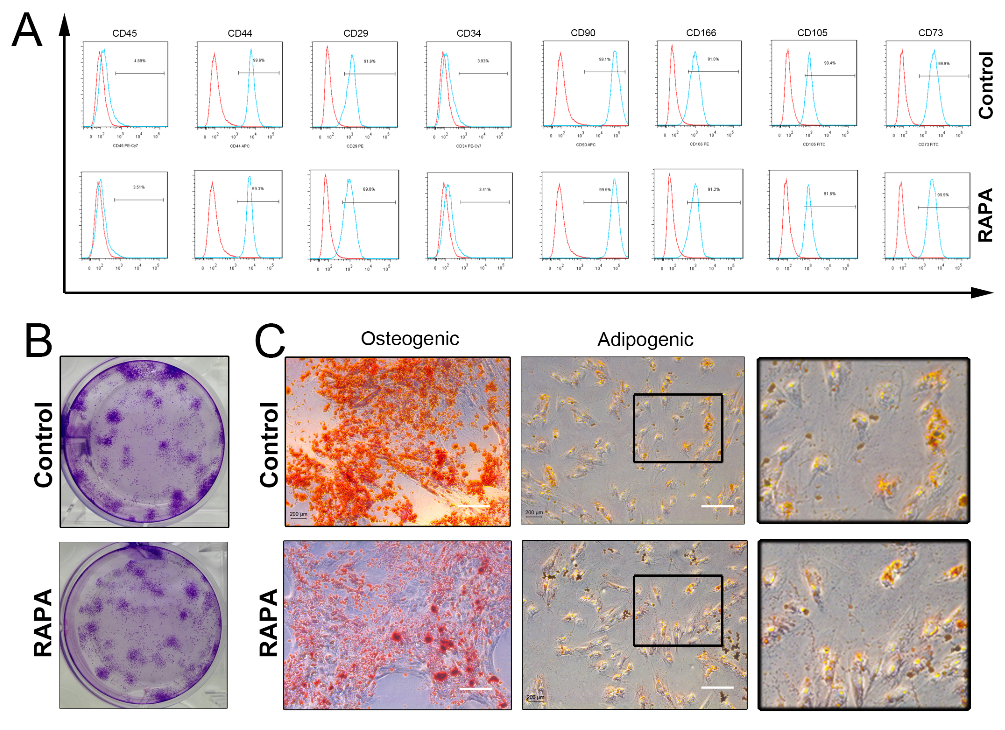
**

**Supplemental Figure 1. Isolation and identification of UC-MSCs and RAPA-preconditioning UC-MSCs.**

(A) Flow cytometric analysis was used to determine cell surface markers, which were positive for CD44, CD29, CD90, CD166, CD105, CD73 and negative for CD45, CD34. (B) Representative images of the colony-forming units of UC-MSCs and RAPA-preconditioning UC-MSCs (P3). (C) Representative images of UC-MSCs and RAPA-preconditioning UC-MSCs staining with Alizarin Red following 21 day of osteogenic induction and representative images of UC-MSCs and RAPA-preconditioning UC-MSCs staining with Oil Red O following 14 days of adipogenic induction.


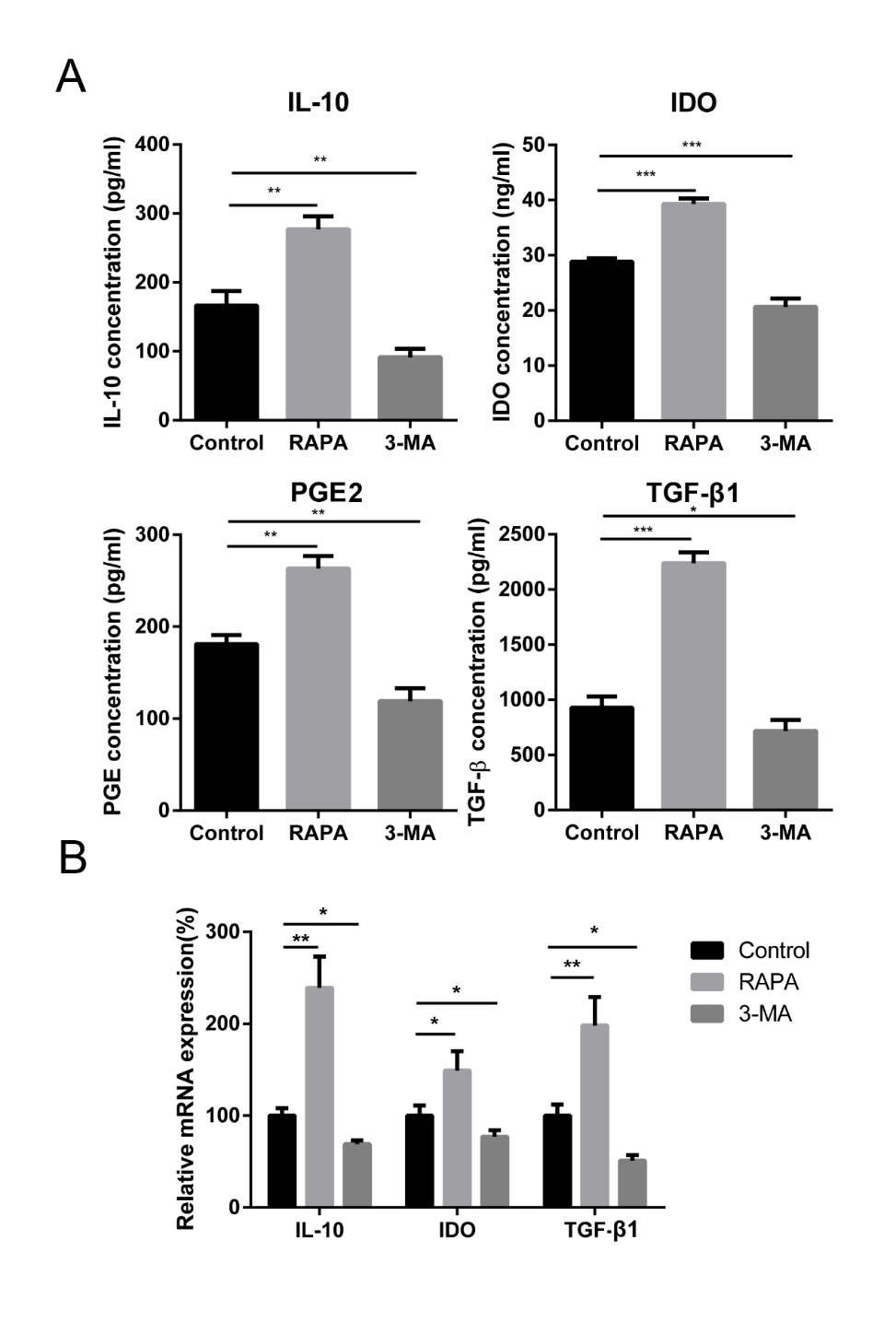


**Supplemental Figure 2. Induced-autophagy strengthened UC-MSCs in anti-inflammatory cytokines release.**

(A) ELISA assay was used to detected the levels of IL-10, IDO, PGE2 and TGF-β1 in UC-MSCs cultured supernatant. Data are presented as mean±SEM (n=5/group). (B) mRNA expressions of IL-10, IDO and TGF-β1 in UC-MSCs was detected. Data are presented as mean±SEM (n=5/group). p<0.05; **, p<0.01; ***, p<0.001. one-way ANOVA analysis.


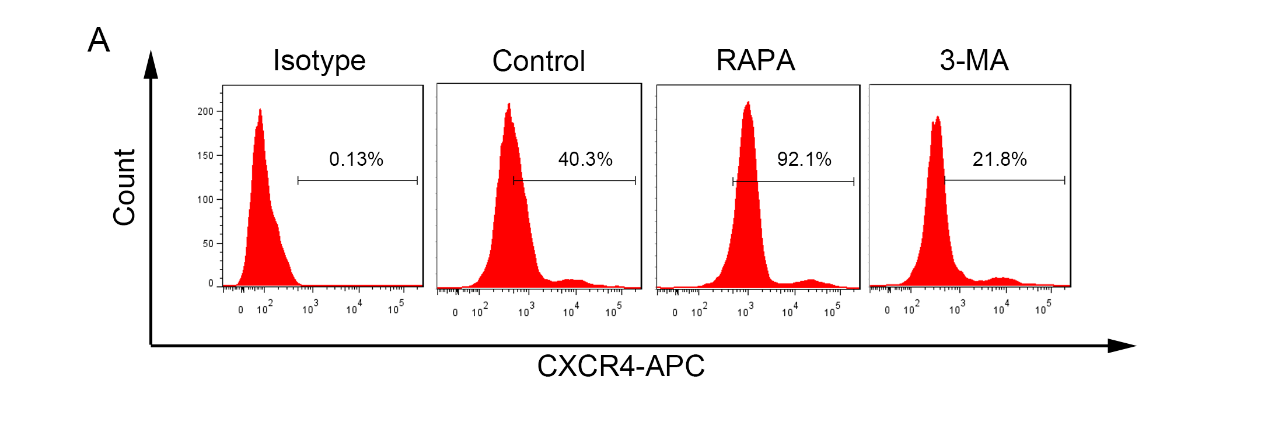


**Supplemental Figure 3. Induced-autophagy upregulated the expression of CXCR4 on UC-MSCs.**

1. Flow cytometry was used to detect the expression of CXCR4 on the surface of UC-MSCs. Five independent samples per group were analyzed.


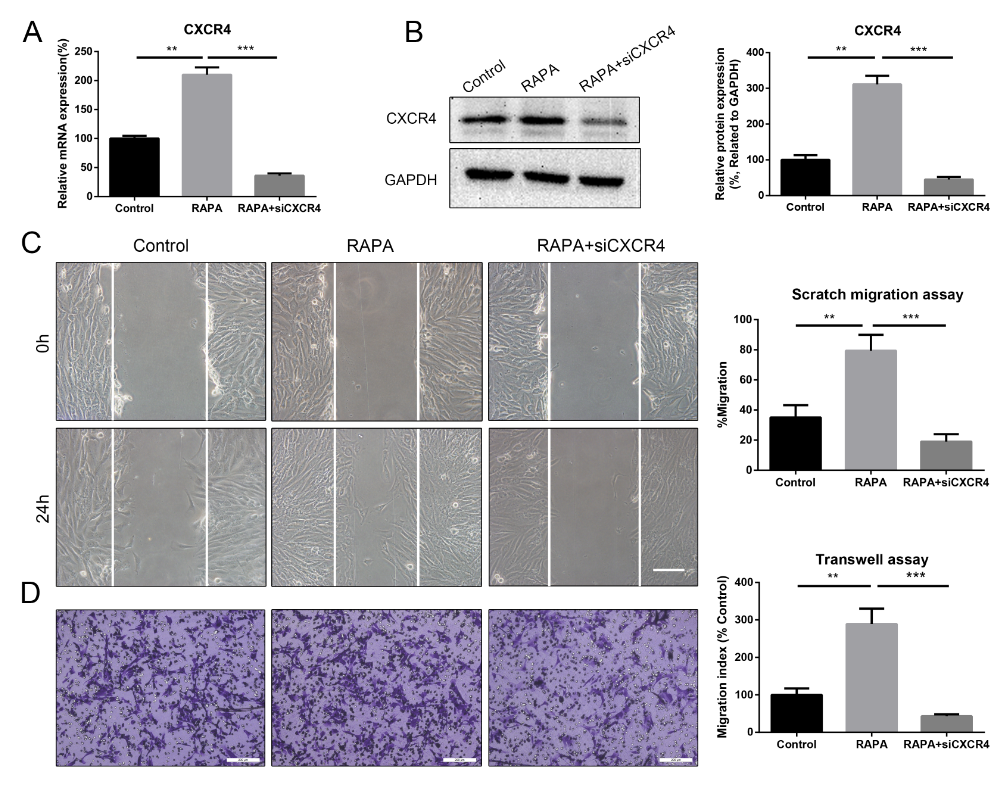


**Supplemental Figure 4. Knockdown of CXCR4 weakened the effect of rapamycin on enhancing UC-MSCs migration.**

The mRNA levels of CXCR4 in each group were detected by real-time PCR. (B) The protein expression of CXCR4 in each group was determined by Western blotting assays. Statistical analysis of the relative density of CXCR4. (C): Representative images of UC-MSCs migration in a scratch migration assay. UC-MSCs were randomly divided into three groups, including control, RAPA and siCXCR4 UC-MSCs+RAPA group (100×). Statistical analyses for the number of migrated cells. (D): Representative images of UC-MSCs migration in a Transwell system. UC-MSCs were randomly divided into three groups, including control, RAPA and siCXCR4 UC-MSCs+RAPA group. Scale bar: 200μm. Statistical analyses for the number of migrated cells. The values of statistical significance between control and treated groups are presented as the mean±SEM. **, p<0.01; ***, p<0.001. one-way ANOVA analysis.


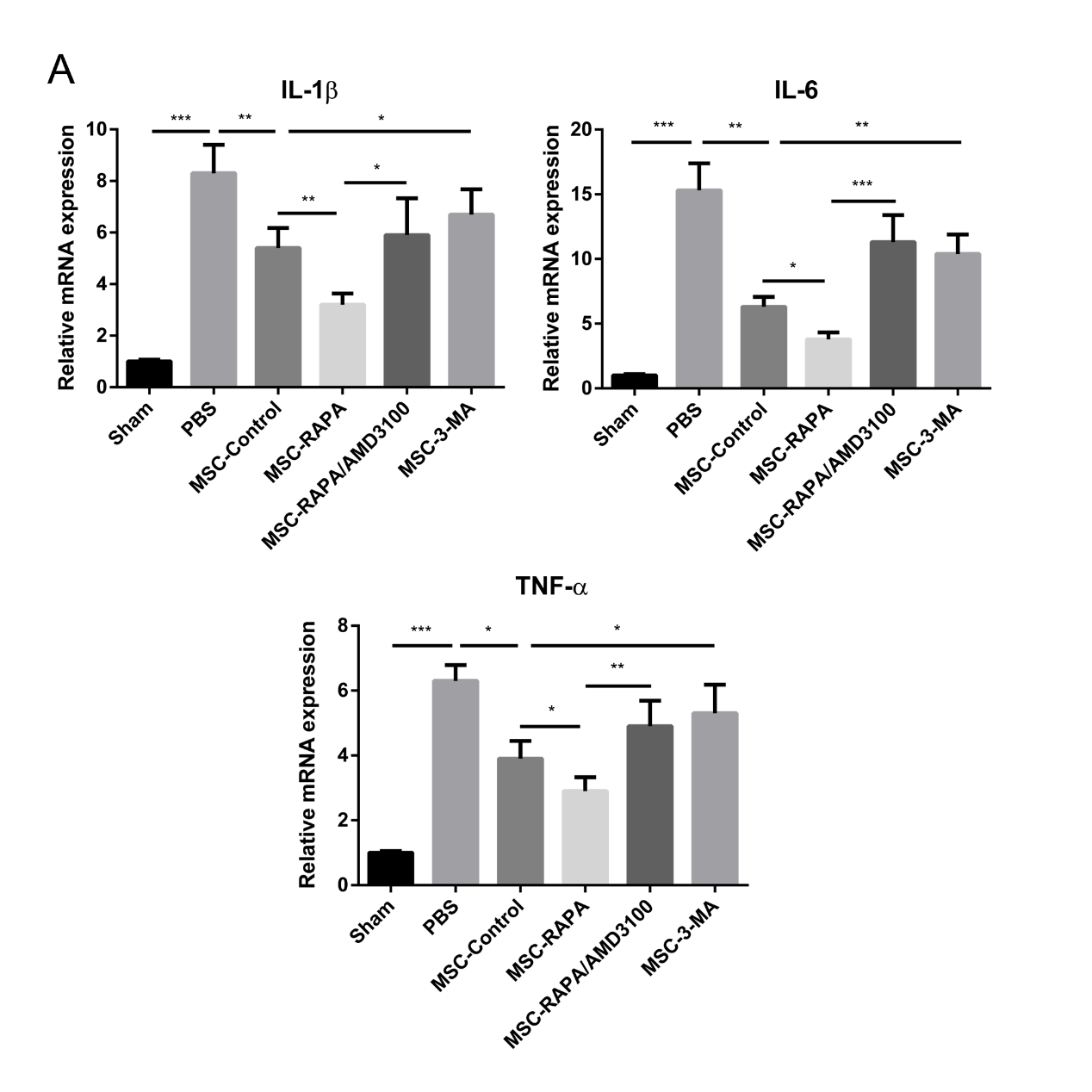


**Supplemental Figure 5. Different levels of autophagy could alter the ability of UC-MSCs in regulating the expression and secretion of cytokine in liver tissue**.

(A) mRNA expression of cytokine in liver tissue, including IL-1β, IL-6 and TNF-α, was evaluated. Data are presented as mean±SEM (n=7 mice/group). *, p<0.05; **, p<0.01; ***, p<0.001. one-way ANOVA analysis.


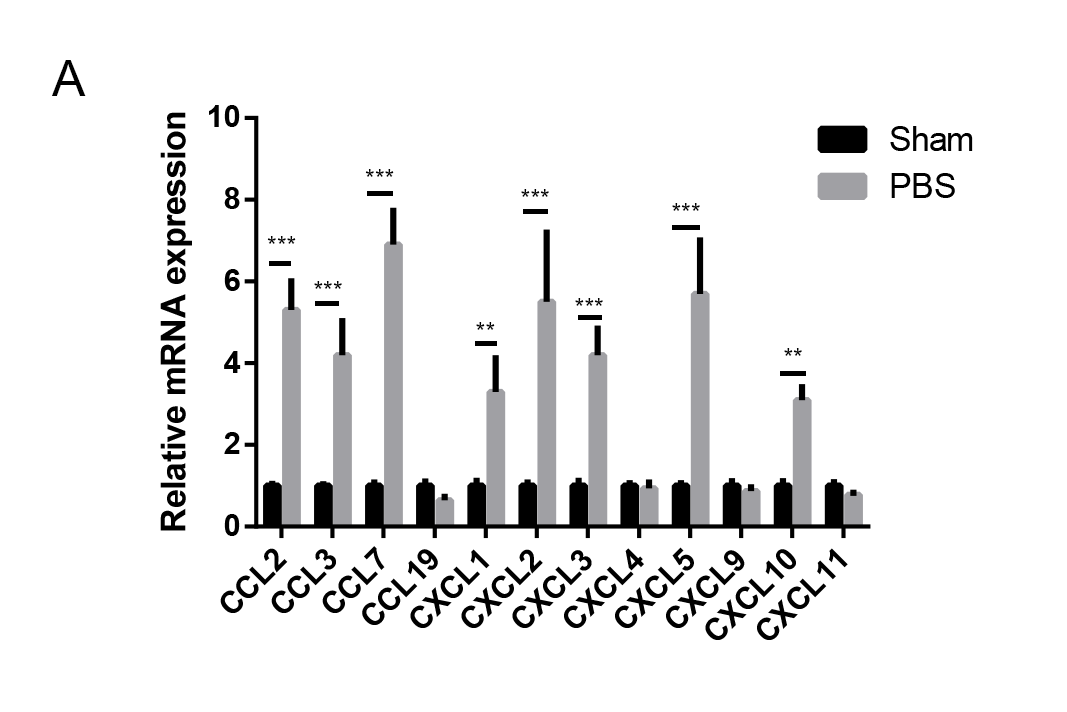


**Supplemental Figure 6. The changes of various chemokines in damage liver after I/R injury.**

(A) Real time-PCT was used to detect the mRNA levels of various chemokines in damage livers after I/R injury. Sample were collected and extracted from Sham group and PBS group. Data are presented as mean±SEM (n=7 mice/group).


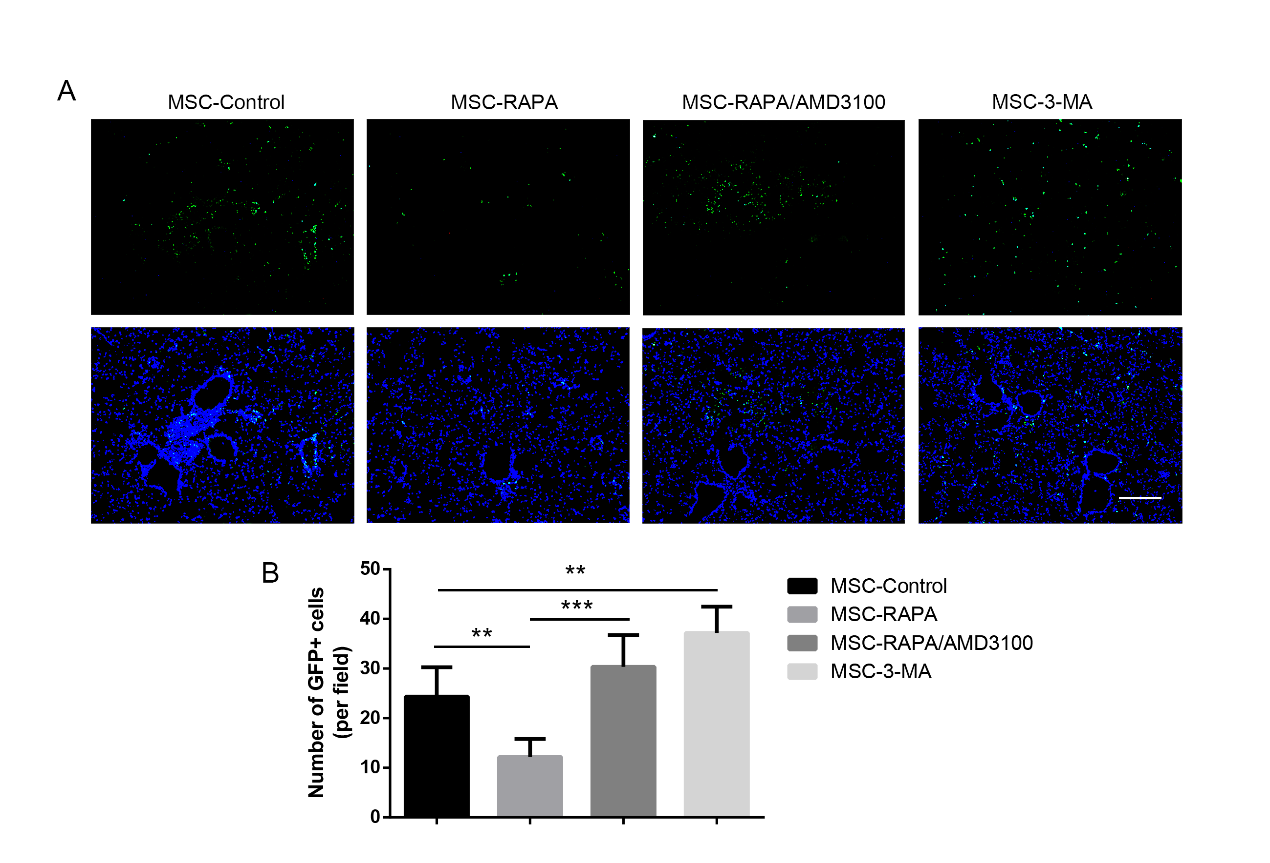


**Supplemental Figure 7. Induction of autophagy reduced the number of UC-MSCs which were blocked in lung**

(A) CellTrackerTM Green CMFDA (green) labelled UC-MSCs were used to examine the count of UC-MSCs engraftment in the lung tissues from each group. Scale bar, 200μm. (B) Quantification of migrated UC-MSCs. Data are presented as mean±SEM (n=7 mice/group). **, p<0.01; ***, p<0.001. one-way ANOVA analysis.
